# Preoperative Serum Albumin Level as a Predictor of Abdominal Wound-Related Complications After Emergency Exploratory Laparotomy

**DOI:** 10.7759/cureus.31980

**Published:** 2022-11-28

**Authors:** Vakulabharanam Naga Rohith, S V Arya, Anita Rani, Raj Kumar Chejara, Ashok Sharma, Jainendra K Arora, Dheer Singh Kalwaniya, Aditya Tolat, Pawan G, Anant Singh

**Affiliations:** 1 General Surgery, Vardhman Mahavir Medical College and Safdarjung Hospital, New Delhi, IND; 2 Biochemistry, Vardhman Mahavir Medical College and Safdarjung Hospital, New Delhi, IND

**Keywords:** surgical site infection (ssi), exploratory laparotomy, organ space surgical site infection, wound dehiscence, preoperative serum albumin level

## Abstract

Background

Serum albumin is generally considered to be a predictor of patients’ nutritional status. Previous studies have used serum albumin to assess postoperative morbidity, mortality, and various other surgical outcomes in cardiac surgeries and elective gastrointestinal surgeries. In this study, we used preoperative serum albumin levels to assess postoperative surgical site wound complications in patients who underwent emergency exploratory laparotomy.

Methodology

Preoperative serum albumin level was observed in 60 patients who underwent emergency exploratory laparotomy due to various pathological conditions and were divided into those with hypoalbuminemia (serum albumin level <3.5 g/dl and >3.5 g/dL). Postoperative surgical site infections, wound dehiscence, and various complications, such as duration of hospital stay, prolonged ileus, the incidence of enterocutaneous fistula, the incidence of anastomotic leak, and 30-day mortality, were assessed.

Results

In our study, about 65% of the patients had hypoalbuminemia. Among them, 56.4% of the patients had surgical site infections according to the Southampton grade, with a statistically significant p-value of <0.001. Moreover, 87.2% of the patients had wound dehiscence according to the World Union Wound Healing Societies Surgical Wound Dehiscence wound grading, with a statistically significant p-value of <0.001. In addition, statistical significance was noted between preoperative hypoalbuminemia and increased postoperative hospital stay, with a p-value of <0.001.

Conclusions

Preoperative serum albumin value is a formidable predictor of postoperative surgical site infections, wound dehiscence, and duration of hospital stay in patients who underwent emergency exploratory laparotomy.

## Introduction

Abdominal surgeries in the emergency are very commonly done across hospitals in India. Although surgical and perioperative improvements have reduced postoperative mortality over the last few decades, postoperative morbidity remains high. In addition to the morbidity patients are exposed to, postoperative complications pose a significant financial burden [1]. The magnitude of the metabolic stress response with the long duration of surgery may contribute to the development of postoperative complications. Early identification of patients with nutritional deficiencies and comorbidities and adequate initial resuscitation may help in reducing the postoperative mortality and morbidity of patients [2-4].

Albumin, a crucial protein, transports hormones, fatty acids, and exogenous medications while also regulating plasma oncotic pressure. Because albumin levels drop during injuries and infection, albumin is referred to as a negative active-phase protein [5]. A maintenance protein called serum albumin is quickly downregulated by inflammatory signals. Low levels of serum albumin are mostly brought on by inflammatory conditions, specifically by high levels of cytokines interleukin-6 (IL-6) and tumor necrosis factor-alpha (TNF-alpha). A common finding in both acute and chronic diseases is hypoalbuminemia. Although new data suggest that increased catabolism is a more frequent reason, hypoalbuminemia in chronic illness has traditionally been attributed to decreased albumin synthesis because of wasting and cachexia. The mechanisms causing hypoalbuminemia in acute conditions are different from those in chronic diseases because capillary leakage into the interstitial space as a result of inflammatory processes is the main source of hypoalbuminemia in acute conditions. Additionally, reduced synthesis, dilution of blood due to fluid administration, renal and intestinal losses due to congestion, and increased catabolism also play a role [6-8].

Preliminary data suggested that albumin levels rapidly dropped after surgery and correlated to outcomes in esophageal and oral cancer, abdominal pancreatic and liver resection/transplant, and cardiac surgeries [6,7]. A previous study suggested that preoperative serum albumin level in patients without certain comorbid diseases is associated with postoperative infectious and surgical complications. Low serum albumin levels can be used as a marker for nutritional deficiency [9]. A serum albumin level >3.5 g/dL suggests adequate protein stores and confers a protective effect through several biological mechanisms. It predicts perioperative morbidity and mortality. Serum albumin level <3.5 g/dL in a stable and well-hydrated patient suggests malnutrition [10]. Further, previous studies suggested that preoperative serum albumin is a good predictor of surgical outcomes in elective gastrointestinal surgeries as well as many other surgeries except cardiac surgeries [11,12].

In 1999, 54,215 cases were studied in 44 tertiary care centers in a prospective observational study on preoperative serum albumin level as a predictor of operative mortality and morbidity. The study included all surgical procedures performed under general, spinal, and epidural anesthesia, with the exception of low-mortality procedures such as ophthalmological procedures, operations on patients who had been enrolled in the study for an index procedure within the previous 30 days, and cardiac surgeries. The study measured 30-day operative mortality and morbidity, finding that a decrease in serum albumin concentrations from greater than 46 g/L to less than 21 g/L was associated with an increase in operative mortality and morbidity. The study found that serum albumin level predicted surgical outcomes better than several other preoperative patient variables [12].

An observational prospective study conducted on preoperative serum albumin level as a predictor of surgical complications after emergency abdominal surgery in a rural tertiary care center in 2019 on 190 patients who underwent emergency abdominal surgery was reported by Sharath Kumar et al. The study assessed multiple postoperative local complications such as surgical site infection, anastomosis breakdown, delayed wound healing, and paralytic ileus. The study concluded that preoperative serum albumin was a good predictor of surgical outcomes after emergency abdominal surgery [13].

There is a paucity of studies examining the role of preoperative serum albumin as a predictor of postoperative complications after emergency abdominal surgeries and hardly any studies have revealed the role of this as a marker of postoperative abdominal wound-related complications after emergency abdominal surgeries. Hence, we conducted this study to evaluate the role of preoperative serum albumin in postoperative surgical site wound complications in patients who underwent emergency exploratory laparotomy.

## Materials and methods

This study was conducted at the Department of General Surgery, Vardhman Mahavir Medical College and Safdarjung Hospital, New Delhi after obtaining approval from the Institutional Ethics Committee (IEC) and the Review Board Committee (approval number: IEC/VMMC/SJH/Thesis/2022-11/CC-147). In this study, we aimed to investigate the value of preoperative serum albumin level as a predictor of postoperative wound-related complications after emergency abdominal surgery.

Study objectives

Primary Objectives

This study aimed to correlate preoperative serum albumin levels with postoperative abdominal wound-related complications till 30 days after surgery. The incidence and grade of surgical site infections were assessed according to the Southampton grading system [14]. The incidence and grade of wound dehiscence were assessed according to the World Union Wound Healing Societies Surgical Wound Dehiscence (WUWHS SWD) grading system [15].

Secondary Objectives

In addition, we aimed to correlate preoperative serum albumin level with postoperative complications such as duration of hospital stay, prolonged ileus, the incidence of enterocutaneous fistula, the incidence of anastomotic leak, and 30-day mortality.

Study design

This observational, prospective study with a sample size of 60 was conducted for 18 months. The sample size was estimated based on a study of the association between preoperative hypoalbuminemia and postoperative wound-site surgical complications in patients [13]. We defined a relevant clinical difference of 20% in surgical complications between the two groups (albumin <3.5 mg/dL and albumin >3.5 mg/dL). We chose a 3% baseline ratio of surgical complication rates in patients with albumin >3.5 mg/dL. Thus, a sample size of 57 patients per group provided a 90% power for detecting a significant difference between the two groups at an alpha level of 0.05 [13].

Inclusion and exclusion criteria

Patients aged 18 years and above who were admitted for emergency abdominal surgery with an Acute Physiology and Chronic Health Evaluation II (APACHE II) score less than or equal to 10 were included in the study [16]. Patients with comorbid illnesses such as kidney disease, liver disease, hypertension, and diabetes mellitus and those who underwent re-exploratory laparotomy within 30 days of the previous surgery were excluded from the study.

A total of 60 patients who underwent emergency abdominal surgery with an APACHE II score of less than or equal to 10 were included in the study. All patients were explained about the study in their native language, and written informed consent was obtained from all patients. A detailed history was taken, and a thorough clinical examination was performed. Relevant biochemical and radiological investigations were done. Data were collected regarding preoperative serum albumin in patients who underwent emergency abdominal surgery in our institution. The incidence and grading of postoperative wound-related complications such as surgical site infection by Southampton grading, wound dehiscence by WUWHS SWD grading, and postoperative prolonged ileus, duration of hospital stay, and certain postoperative complications, such as an anastomotic leak, enterocutaneous fistula, and 30-day mortality, were assessed. The correlation between preoperative serum albumin level and postoperative complications was evaluated.

For this study, normal albumin levels were considered to be 3.5-5.5 g/dL [9]. Hypoalbuminemia was defined as serum albumin levels <3.5 g/dL [13]. Surgical site infection was considered to be present if it met the European Centre for Disease Prevention and Control (CDC) criteria [17]. Surgical site infection was graded according to the Southampton grading system [14]. SWD was considered to be present if there was the separation of the margins of a closed surgical incision made in the skin, with or without exposure or protrusion of underlying tissue, organs, or implants. Separation may occur at single or multiple regions, or involve the full length of the incision, and may affect some or all tissue layers. A dehisced incision may display clinical signs and symptoms of infection. SWD was identified and graded according to the WUWHS SWD grading system [15].

Data analysis

Statistical testing was conducted using SPSS Statistics version 21.0 (IBM Corp., Armonk, NY, USA). Continuous variables were presented as mean ± standard deviation (SD) or median (interquartile range, IQR) for non-uniformly distributed data. Categorical variables were expressed as frequencies and percentages. Uniformly distributed continuous variables between the groups were compared using Student’s t-test. Nominal categorical data between the groups were compared using the chi-square test or Fisher’s exact test as appropriate. Non-normally distributed continuous variables were compared using the Mann-Whitney U test. For all statistical tests, a p-value less than 0.05 indicated a statistically significant difference.

## Results

**Figure 1:**
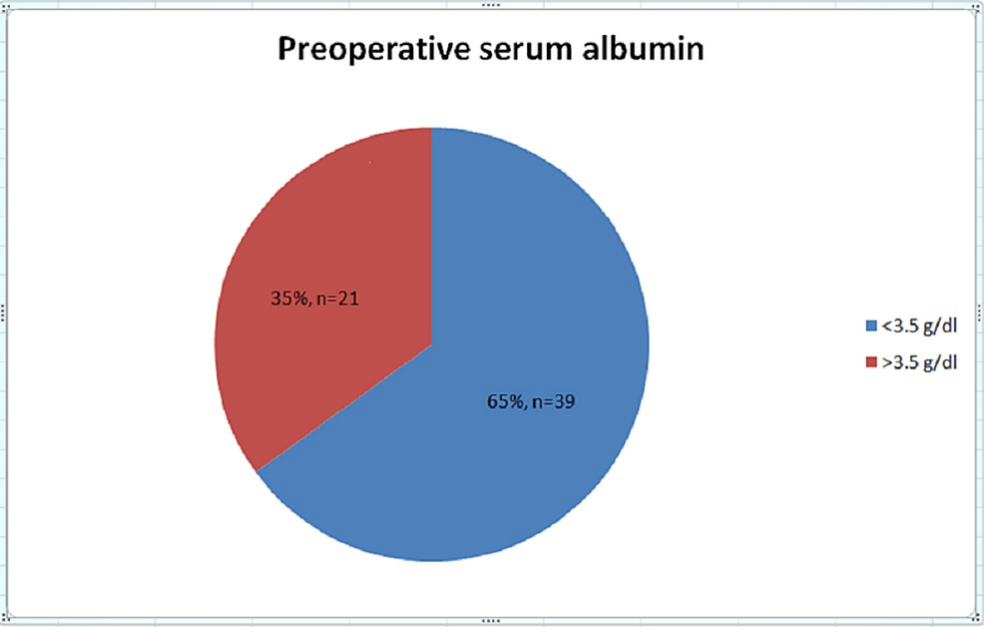
Pie chart showing the distribution of preoperative serum albumin levels in the study population.

**Figure 2:**
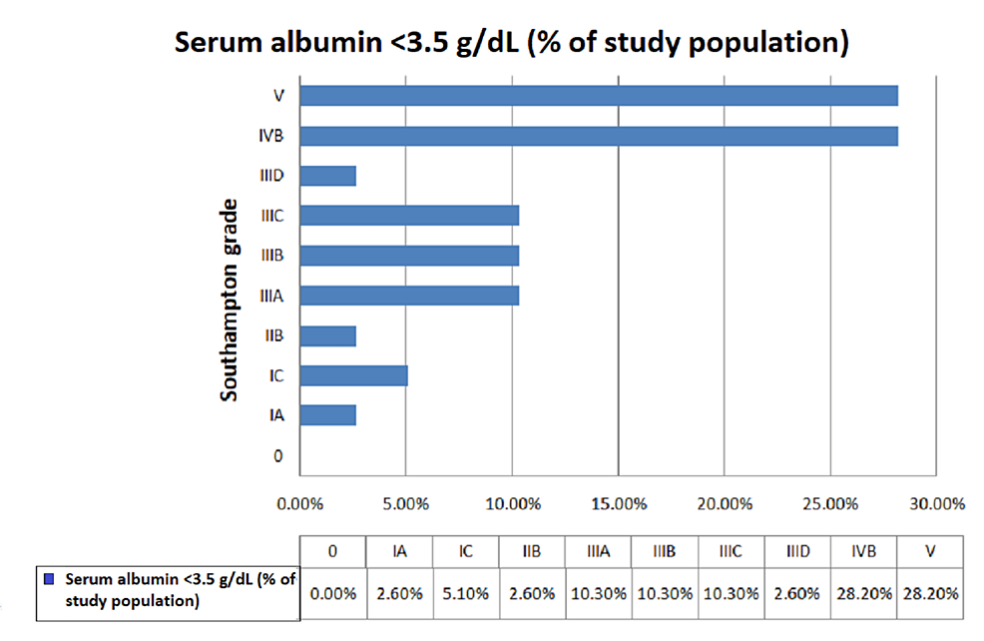
Percentage distribution of surgical site infections according to Southampton grading in hypoalbuminemia patients.

**Figure 3:**
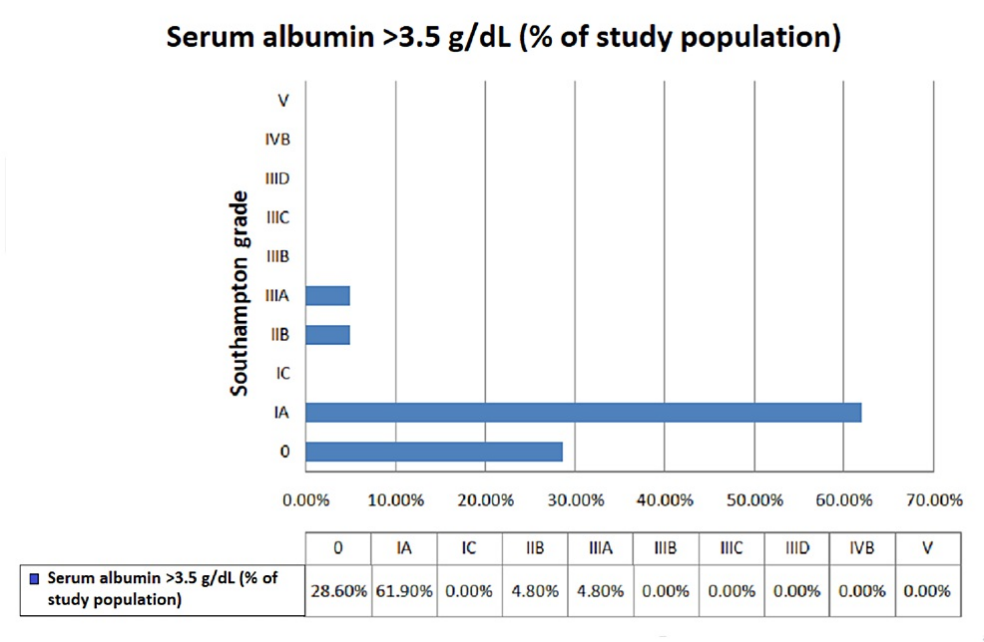
Percentage distribution of surgical site infections according to Southampton grading in patients with normal serum albumin levels.

**Figure 4:**
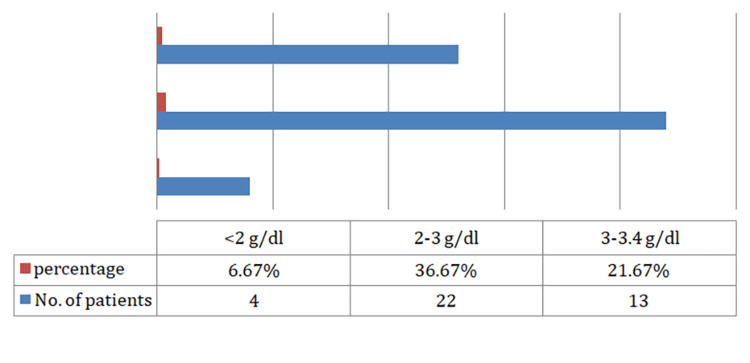
Distribution of preoperative hypoalbuminemia patients.

We observed that the majority of the patients who underwent emergency abdominal surgery were between the ages of 21 and 30 years (38.3%), with a mean age of 34.2 years. In our study, the majority were males (61.7%), followed by females (38.3%). We observed that the majority of patients underwent emergency exploratory laparotomy due to hollow viscus perforation (41.8%), followed by intestinal obstruction (23.3%). Overall, 65% of the study population had preoperative hypoalbuminemia (serum albumin level <3.5 g/dL) (Figure 1). Patients with preoperative hypoalbuminemia had significant postoperative surgical site infection graded as IVB (28.2%) and V (28.2%) according to the Southampton grading. However, among patients with preoperative serum albumin between 3.5 and 5.5 g/dL, the majority were graded I A (61.9%), followed by normal healing (28.6%), with a statistically significant p-value of <0.001 (Figures 2, 3). Among patients who had preoperative serum albumin levels less than 2 g/dL (6.67%), all patients had complete wound dehiscence (Figure 4).

**Figure 5:**
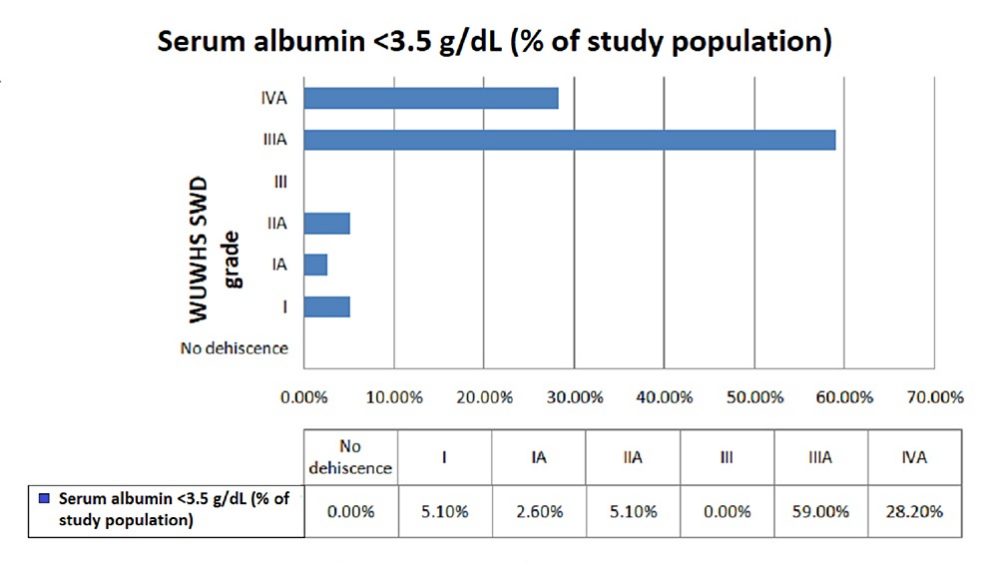
Distribution of wound dehiscence in patients with hypoalbuminemia according to WUWHS SWD grading. WUWHS SWD: World Union Wound Healing Societies Surgical Wound Dehiscence

**Figure 6:**
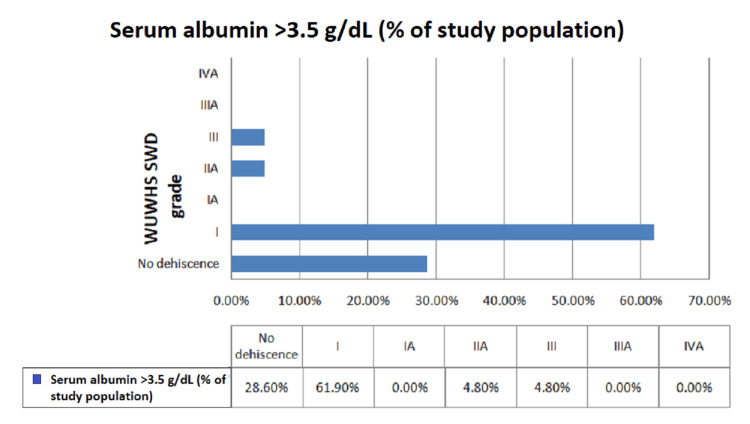
Distribution of wound dehiscence in patients with normal serum albumin level according to WUWHS SWD grading. WUWHS SWD: World Union Wound Healing Societies Surgical Wound Dehiscence

**Figure 7:**
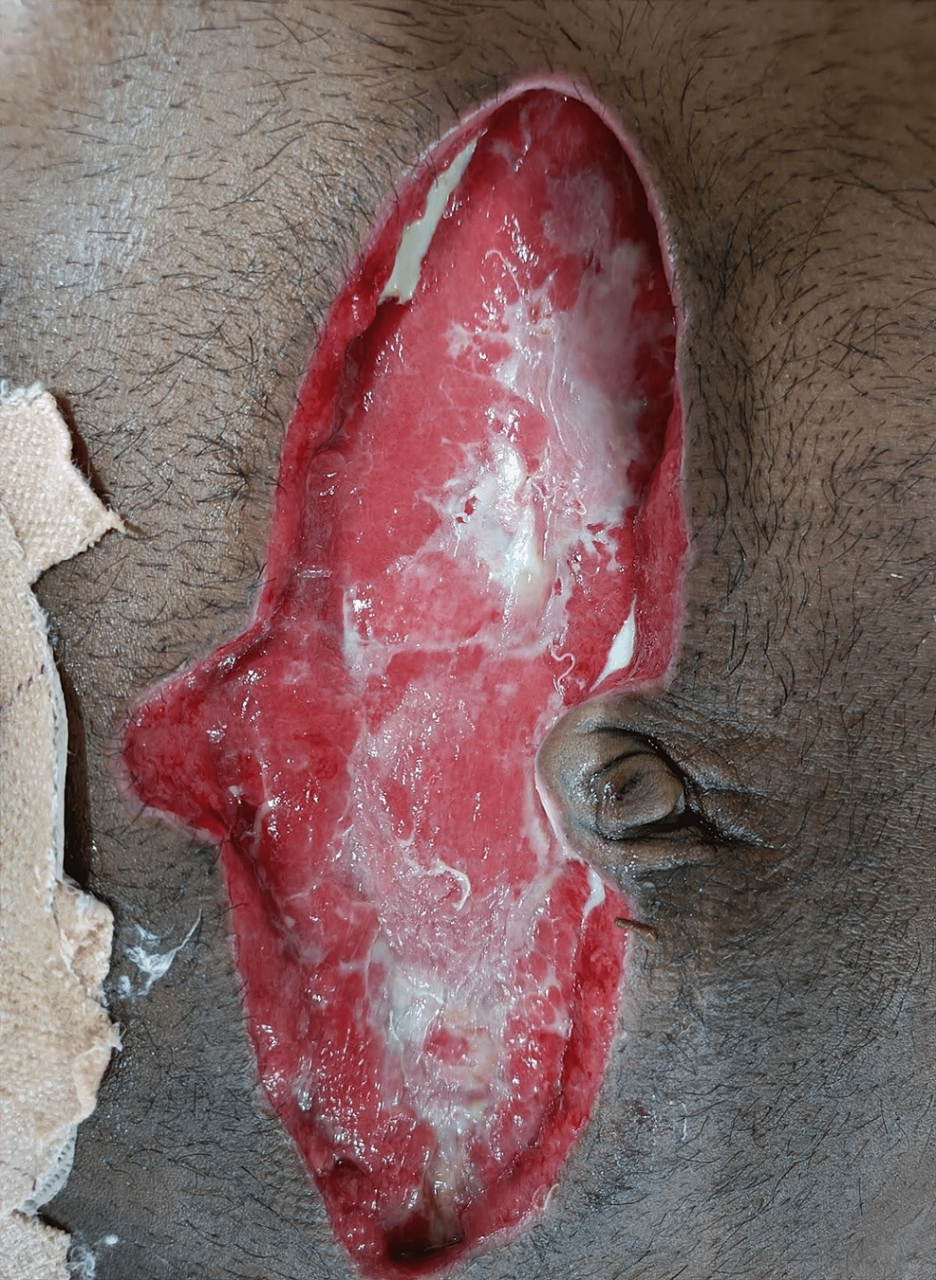
A clinical photograph of a patient with complete wound dehiscence.

In addition, patients with preoperative hypoalbuminemia had significant deep wound infections and wound dehiscence and according to the WUWHS SWD, with grade IIIA (59.2%) and grade IVA (28.2%) (Figure 5). However, among those with preoperative serum albumin levels ranging between 3.5 and 5.5 g/dL, the majority (61.9%) were graded I, followed by no dehiscence (28.6%) according to WUWHS SWD grading (Figure 6), with a statistically significant p-value of <0.001. Prolonged hospital stay was observed in patients postoperatively who had low preoperative serum albumin levels, which was statistically significant. Only three (7.7%) patients had prolonged ileus postoperatively among those who had preoperative serum albumin levels <3.5 g/dL, which was statistically insignificant. Only one patient had an anastomotic leak and enterocutaneous fistula postoperatively, i.e., 2.6% each among patients who had preoperative serum albumin levels <3.5 g/dL, which was also statistically insignificant. The reason for these complications could be the location of the perforation, which was the duodenum, and late presentation to the emergency room after trauma. In our study, no patient had mortality within 30 days. Figure 7 shows the complete wound dehiscence of patients included in this study (Figure 7).

## Discussion

In this study, 60 patients were included with ages ranging from 18 to 77 years. The mean age of the patients was 34.2 years. Overall, 38.3% of the study population was aged 21-30 years. The greater burden of patients who underwent surgery was between 21 and 30 years. However, in contrast to our study, in the study by Gibbs et al. [12], the median age was 61 years. In another study by Bhandari et al. [11], the median age was 50 years. The patients included in our study were mostly in the age group of 21-30 years, which could be due to the exclusion of comorbidities such as hypertension, diabetes mellitus, liver disease, and kidney disease which are common in the elderly age group.

In our study, of the 60 patients, 38.3% were females and 61.7% were males. Hence, the majority of the patients who underwent emergency surgery were males. Similar results were reported in a previous study by Sharath Kumar et al. [13], in which 61.5% were males and 38.5% were females. In another study by Bhandari et al. [11], 66% were males and 34% were females. Males were more commonly involved in road traffic accidents compared to females because males are at high risk while traveling compared to females who mostly stay at home in a developing country like India. This could be the reason for more male patients undergoing exploratory laparotomy in trauma cases. Another reason could be the presence of risk factors usually found in males rather than females, such as alcoholism, smoking, and drug abuse, which are usually responsible for increased trauma and conditions such as pre-pyloric perforation, hollow viscus perforation, and ruptured liver abscess.

In our study, the majority of the patients who underwent emergency exploratory laparotomy were because of hollow visceral perforation (41.8%), followed by acute intestinal obstruction (23.3%), and hemoperitoneum (16.7%). Similar results were noted in the study by Sharath Kumar et al. [13] where indications for emergency surgery were majorly due to hollow visceral perforation (45.8%), followed by intestinal obstruction (23.2%).

Among the 60 patients, preoperative serum albumin levels ranged from 1.8 to 4 g/dL, with a mean of 3.12 g/dL and a median of 3.1 g/dL. Overall, 65% of the patients had hypoalbuminemia (serum albumin level <3.5 g/dL), and 35% of patients had normal serum albumin levels (>3.5 g/dL to 5.5 g/dL). Similar results were noted in the study by Sharath Kumar et al. [13], in which 63.1% of the patients had hypoalbuminemia and 36.9% of the patients had normal albumin levels.

In our study, among the 39 patients who had hypoalbuminemia, four (6.67%) patients had preoperative serum albumin levels less than 2 g/dL, 22 (36.67%) patients had preoperative serum albumin levels of 2-3 g/dL, and 13 (21.67%) patients had serum albumin level of 3-3.4 g/dL. Of the patients with preoperative albumin levels less than 2 g/dL, all patients had complete wound dehiscence. In the previous study by Sharath Kumar et al. [13], patients with preoperative serum albumin levels of less than 2 g/dL had significant postoperative complications and mortality.

Among patients with preoperative serum albumin <3.5 g/dL, the majority were graded V (28.2%) and IVB (28.2%) according to the Southampton grading system. However, among patients with preoperative serum albumin between 3.5 and 5.5 g/dL, the majority were graded I A (61.9%), followed by normal healing (28.6%). Hence, there was a statistically significant difference in the proportion of Southampton grading according to preoperative serum albumin levels (p < 0.05). In the study by Sharath Kumar et al. [13], the incidence of surgical site infections was 59.1% in patients with preoperative hypoalbuminemia, which was also statistically significant and similar to our study. In another study conducted by Gibbs et al. [12], the incidence of deep wound infections was 5.9% and the incidence of superficial wound infections was 4.4%, with statistically significant p-values of <0.001.

Among patients with preoperative serum albumin <3.5 g/dL, the majority of the patients were graded IIIA (59%) followed by IVA(28.2%). There was a statistically significant difference in the proportion of WUWHS SWD grade according to preoperative serum albumin (p < 0.05). However, in the study by Sharath Kumar et al. [13], the incidence of deep wound space infections was 3.2% in patients with hypoalbuminemia. In another study by Gibbs et al. [12], the incidence of deep wound infections and wound dehiscence was 5.9% and 2%, respectively, in patients who had preoperative hypoalbuminemia, with statistically significant p-values of <0.001.

The duration of hospital stay in the study population ranged from five to 80 days, with a mean of 11.08 (±10.48) days. Among patients with preoperative serum albumin <3.5 g/dL, 23.1% of the patients stayed in the hospital for ≤7 days, 51.3% stayed in the hospital for 8-14 days, and 25.6% stayed in the hospital for >14 days. There was a statistically significant difference in the proportion of duration of hospital stay according to preoperative serum albumin (p < 0.05). In a previous study by Amavizca et al. [18], low serum albumin level shortly after admission was used to predict prolonged hospital stay in younger burn patients. The study revealed that among burn patients aged less than or equal to 40 years, 16% sustained a prolonged hospital stay, i.e., more than three weeks. Moreover, using serum albumin as a sole predictor, 87.5% of patients with prolonged stay were correctly predicted, which was statistically significant.

In our study, only three (5%) patients had prolonged ileus. Among patients with preoperative serum albumin <3.5 g/dL, three (7.7%) patients had prolonged ileus. However, among patients with preoperative serum albumin between 3.5 and 5.5 g/dL, none had prolonged ileus, with a statistically insignificant difference in the proportion of prolonged ileus according to preoperative serum albumin (p > 0.05). Similar results were reported in a previous study by Sharath Kumar et al. [13], with the incidence of prolonged ileus at 4.3% in patients with preoperative hypoalbuminemia. In general, the presence of preoperative hypoalbuminemia causes postoperative prolonged ileus because of bowel edema; however, in our study, there was no statistical significance between preoperative hypoalbuminemia and postoperative prolonged ileus.

In our study, only one (1.7%) patient had an anastomotic leak. Among patients with preoperative serum albumin <3.5 g/dL, one (2.6%) patient had an anastomotic leak. However, among patients with preoperative serum albumin between 3.5 and 5.5 g/dL, none had an anastomotic leak. Hence, there was a statistically insignificant difference in the proportion of anastomotic leaks according to preoperative serum albumin levels (p > 0.05). However, in contrast to our study, in the study by Sharath Kumar et al. [13], the incidence of anastomotic leak in patients with preoperative hypoalbuminemia was 28%, with a statistically significant p-value of <0.005.

In our study, only one (1.7%) patient had an enterocutaneous fistula. Among patients with preoperative serum albumin <3.5 g/dL, one (2.6%) patient had an enterocutaneous fistula. However, among patients with preoperative serum albumin between 3.5 and 5.5 g/dl, none had an enterocutaneous fistula. Hence, there was a statistically insignificant difference in the proportion of enterocutaneous fistulas according to preoperative serum albumin (p > 0.05). However, in contrast to our study, in the study by Sharath Kumar et al. [13] the incidence of enterocutaneous fistula in patients with preoperative hypoalbuminemia was 5.4%, which was significant. However, in our study, for the patient who had both an anastomotic leak and an enterocutaneous fistula, the preoperative serum albumin level was 3.2 g/dL. The cause of these postoperative complications could be the site of perforation, which was the duodenum, and late presentation to the emergency room, i.e., eight days after the trauma.

According to our study, there is a significant association between preoperative hypoalbuminemia and postoperative wound-related complications such as surgical site infection and wound dehiscence which were measured by Southampton grading and WUWHS SWD grading, respectively. There was also a significant association between preoperative hypoalbuminemia and postoperative prolonged hospital stay.

Study limitations

The half-life of serum albumin is 19 days, and it varies accordingly in acute and chronic illnesses, sepsis, and injuries. Preoperative serum albumin level in our study was not able to predict postoperative prolonged ileus, anastomotic leak, enterocutaneous fistula, and postoperative mortality within 30 days. Serum albumin level also alters in many other acute and chronic conditions. This study conducted in our hospital included a limited population and was conducted in a single center. Multicentric analyses with more participants are required to evaluate the preoperative serum level as a sole predictor of postoperative surgical site infections.

## Conclusions

Preoperative serum albumin level can be used as a predictor of postoperative wound-related complications in emergency exploratory laparotomy patients. Moreover, it can predict the progression of the patient and the prolonged hospital stay of the patient postoperatively. However, in our study, there was no significant association between preoperative hypoalbuminemia and postoperative complications such as anastomotic leak, enterocutaneous fistula, and postoperative 30-day mortality. Further studies with appropriate sample sizes are needed for further evaluation of preoperative hypoalbuminemia and the above-mentioned complications.

## Appendices

**Table 1:** The WUWHS SWD grading system. SSI: surgical site infection; WUWHS SWD: World Union Wound Healing Societies Surgical Wound Dehiscence

Grade	Description
I	Dermal layer only involved; no visible subcutaneous fat. No clinical signs and symptoms of infection
Ia	As Grade 1 plus clinical signs and symptoms of infection (e.g., superficial incisional SSI)
II	Subcutaneous layer exposed; fascia not visible. No clinical signs and symptoms of infection
IIa	As Grade 2 plus clinical signs and symptoms of infection (e.g., superficial incisional SSI)
III	Subcutaneous layers and fascia exposed. No clinical signs and symptoms of infection
IIIa	As Grade 3 plus clinical signs and symptoms of infection (e.g., deep incisional SSI)
IV	Any area of fascial dehiscence with organ space, viscera, implant, or bone exposed. No clinical signs and symptoms of infection
IVa	As Grade 4 plus clinical signs and symptoms of infection (e.g., organ/space SSI)

## References

[1] Vonlanthen R, Slankamenac K, Breitenstein S (2011). The impact of complications on costs of major surgical procedures: a cost analysis of 1200 patients. Ann Surg.

[2] Thorell A, Nygren J, Ljungqvist O (1999). Insulin resistance: a marker of surgical stress. Curr Opin Clin Nutr Metab Care.

[3] Mantziari S, Hübner M, Coti-Bertrand P, Pralong F, Demartines N, Schäfer M (2015). A novel approach to major surgery: tracking its pathophysiologic footprints. World J Surg.

[4] Hall R (2013). Identification of inflammatory mediators and their modulation by strategies for the management of the systemic inflammatory response during cardiac surgery. J Cardiothorac Vasc Anesth.

[5] Issangya CE, Msuya D, Chilonga K (2020). Perioperative serum albumin as a predictor of adverse outcomes in abdominal surgery: prospective cohort hospital based study in Northern Tanzania. BMC Surg.

[6] Labgaa I, Joliat GR, Kefleyesus A, Mantziari S, Schäfer M, Demartines N, Hübner M (2017). Is postoperative decrease of serum albumin an early predictor of complications after major abdominal surgery? A prospective cohort study in a European centre. BMJ Open.

[7] Keller U (2019). Nutritional laboratory markers in malnutrition. J Clin Med.

[8] Jäntti T, Tarvasmäki T, Harjola VP (2019). Hypoalbuminemia is a frequent marker of increased mortality in cardiogenic shock. PLoS One.

[9] Paocharoen Paocharoen, Mingphreudhi S, Lertsithichai P, Euanorasetr C (2003). Preoperative serum albumin level and postoperative septic complications. Thai J Surg.

[10] Sharma L, Purohit PM, Pipal D, Kothari S, Srivastava H, Soni A (2017). Serum albumin as predictor of post-operative morbidity and mortality. Int Surg J.

[11] Bhandari TR, Shahi S, Bhandari RS, Lakhey PJ (2016). Preoperative serum albumin level as a predictor of perioperative outcome in patients undergoing major gastrointestinal surgery. J Soc Surg Nepal.

[12] Gibbs J, Cull W, Henderson W, Daley J, Hur K, Khuri SF (1999). Preoperative serum albumin level as a predictor of operative mortality and morbidity: results from the National VA Surgical Risk Study. Arch Surg.

[13] Sharath Kumar V, Prakash DG, Pottendla VK (2019). Preoperative serum albumin level as a predictor of surgical complications after emergency abdominal surgery. Int Surg J.

[14] Bailey IS, Karran SE, Toyn K, Brough P, Ranaboldo C, Karran SJ (1992). Community surveillance of complications after hernia surgery. BMJ.

[15] (2018). World Union of Wound Healing Societies (WUWHS) Consensus Document. Surgical wound dehiscence: improving prevention and outcomes. https://www.woundsinternational.com/resources/details/consensus-document-surgical-wound-dehiscence-improving-prevention-and-outcomes.

[16] Knaus WA, Draper EA, Wagner DP, Zimmerman JE (1985). APACHE II: a severity of disease classification system. Crit Care Med.

[17] (2017). European Centre for Disease Prevention and Control. Surveillance of surgical site infections in European hospitals - HAISSI protocol. https://www.ecdc.europa.eu/en/publications-data/surveillance-surgical-site-infections-and-prevention-indicators-european.

[18] Amavizca K, Yang S, Idicula A, Mata A, Dissanaike S (2016). Lower serum albumin shortly after admission predicts prolonged hospital stay in younger burn patients. J Burn Care Res.

